# Thoughts on the Future Medical Care Pattern of Pediatrics in China Based on the Outbreak of COVID-19

**DOI:** 10.1017/dmp.2020.413

**Published:** 2020-10-26

**Authors:** Hui Liu, Li Wang

**Affiliations:** Department of Pediatrics, Daping Hospital, Army Medical University, Chongqing, China

**Keywords:** COVID-19, medical care pattern, pediatrics

## Abstract

The outbreak of pneumonia known as *coronavirus disease* (COVID-19) has occurred in China since December 2019 and spread rapidly across the world. Pediatric medical workers have a serious imbalance doctor–patient ratio in China; they have accumulated experience during the fight against COVID-19; however, some flaws were revealed in their current medical system. Meanwhile, these problems were also reported in other countries. Thus far, the outbreak of COVID-19 is still rampant across the world. The experience from anti-COVID-19 could be useful and teach us to provide better medical services for Chinese children and prepare for similar public emergencies in the future. Furthermore, it also provides guidance for pediatric medical staff in managing COVID-19 in other developing countries.

Although victory has been declared against the initial wave of the coronavirus disease (COVID-19) epidemic in China, the current deficiencies in the pediatric medical services had brought enormous pressure to both the Chinese pediatric medical staff and family members with children. At the beginning of the outbreak of COVID-19, parents were scared due to the high contagion of severe acute respiratory syndrome coronavirus 2 (SARS-CoV-2) and indistinguishable symptoms between a common viral infection and SARS-CoV-2. A rapid increase of children into the pediatric clinics spread anxiety and fear among the parents.

To avoid excessive patients gathering in pediatric clinics, many medical institutions offered online services by various applications, WeChat, official websites, and other Internet platforms during the COVID-19 epidemic.^1^ With time and development of the online medical facilities, the number of pediatric outpatients in the majority of medical institutions has decreased significantly (by 83% in our hospital) compared to that during the early period of the COVID-19 epidemic. The online practice has dramatically reduced the pediatric outpatients, the risk of SARS-CoV-2 transmission and the burden of pediatric medical staff, as well as parents’ anxieties.

However, there are still some limitations: Some examinations are difficult to be performed remotely. Most of the online medical platforms are mainly focused on consultation, which reduces standardized procedures. Moreover, some clinicians are reluctant to participate because of considering medical disputes or time, and some patients are also unwilling to avail online medical care due to trust and privacy.^2^

Based on the these facts and accumulated experience during the COVID-19 epidemic, we provide some recommendations for Chinese online medical services:

First, improve the online medical services system: Standardized training and education of medical workers using online medical care should be conducted.^3^ Moreover, the third-party online medical platform must be qualified for medical services, including online electronic prescription, online pharmacy, and other relevant qualifications. Furthermore, the criteria for online medical staff must be strict and conform to the national regulations.

Second, strengthen the combination of online and offline medical services: Online service is a complement to offline medicine; higher online popularity is conducive for the transition of patients from online to offline.^4^ Appointments, registration, and payment managed via online channels are effective approaches to relieve the pressure of medical services and regional limitation.^4^ Patients could go to hospitals during specified periods according to their appointment times to avoid overcrowding and cross-infection instead of waiting in the hospital for a long time. Also, evaluating the severity of diseases through online consultations during a non-emergency is feasible. Common diseases and routine health care should be managed in primary hospitals or through online medical services. Medical referrals can be carried out in exceptional circumstances when they are required.

## CONCLUSION

A center-hospital model is unfeasible during the COVID-19 epidemic.^5^ A combination of the online and offline medical models could be more valuable for providing better medical services for Chinese children and managing similar public emergencies in the future, as well as providing guidance for pediatric medical staff in other developing countries against COVID-19.
